# The Plague at Dunster in 1645: Remarkable Sanitary Provision

**Published:** 1857-04

**Authors:** A. Haviland


### THE PLAGUE AT DUNSTER IN THE YEAR 1645: REMARKABLE SANITARY REGULATION ADOPTED BY THE INHABITANTS DURING ITS PREVALENCE.*

As Dunster is the town mentioned by Sydenham, in his account of the Plague of the years 1665 and 1666, where a peculiar and somewhat successful mode of treatment of this dreadful disease was adopted, it claims a place in the history of medicine, as well as in that of the eventful period, when both intestine war and pestilence distracted the minds of the sovereign and his subjects.

In a ramble during August, 1855, through the beautiful district, where the Quantock and the Brendon Hills lie side by side, sheltering the picturesque villages which nestle in their valleys, and give life to the alluvial plain bordered by Minehead, Dunster, and Blue Anchor, we were induced to make some inquiries upon a subject of so much interest to the medical profession. We accordingly visited the priory church of Dunster, where we were allowed to make a thorough search of the parish register, an authority that lent us some aid, but which, we regret to say, in these days when Archæological Societies are so flourishing, had been sadly neglected. As our visit took place immediately after the Somersetshire Society had held its congress at Dunster, we naturally expected to find this, the most ancient and important document in the parish, dusted and put into its proper place. Our anticipations were not realised; it was a long time before we could find the register wanted, and when it was found, we discovered that the devastations of time, the worm, civil war, and neglect, had rendered this public record almost useless. These defects in the said register are mentioned only because they considerably interfered with our investigation.

We will first direct attention to the passage in Sydenham, where he devotes considerable space to the discussion of the merits of blood-letting in the treatment of the plague. After quoting many authors, who held that bleeding in pestilential fever was the proper remedy, and deprecating the abstraction of small quantities of blood, which he, with Leonardus Botallus, considers highly injudicious, whilst a full depletion has the

---

* The Works of Thomas Sydenham, M.D. Translated by R. G. Latham, M.D., for the Sydenham Society. 1848. p. 110 et seq.
Clarendon's History of the Rebellion, vol. ii, p. 368.
The Parish Register of Dunster for the year 1645.
Savage's History of the Hundred of Carhampton. pp. 412.
Proceedings of the Somersetshire Archæological and Natural History Society during 1855.

contrary effect, Sydenham thus writes: "I will relate a fact that occurred in our own island a few years ago (1645); it is one of great rarity, and not foreign to the subject. . . . Amongst the other calamities of the civil war, which afflicted our unhappy country, the plague made in many places its ravages. Amongst others, it attacked Dunster, in the county of Somerset. It had been introduced from without; some of the common soldiers it killed at once; plague spots having suddenly broken out. Others also it attacked. There was a certain surgeon, who had returned from a long travel in foreign parts. He was serving as a common soldier; he earnestly entreated the governor to let him do his best for the relief of his fellow soldiers, who were seized with this terrible disease. The governor gave him leave. He bled the patients straight off, the moment the disease seized them, and before any tumour appeared. He took an enormous quantity of blood, keeping on until they were unable to stand on their feet; they stood whilst they were bled: it was done in the open air. There was no vessel to catch or measure the blood; the soil served for basin. He sent them after this to lie down in their tents; he used no remedy beyond bleeding. He treated a great number in this manner; strange to say, not one died. I had this from a man of equal probity and truth; Mr. Francis Wyndham, captain in the army, being my informant."

This passage shews that the plague was at Dunster, and that it was brought there from without. The port of Minehead, where this disease in 1645 slew five times the usual number of the inhabitants, was the inlet through which it travelled to Dunster, where it evidently was not suspected to exist, for Lord Digby signified his Majesty's (Charles I.) wish that the Prince should leave Bristol, where the plague was very fatal, and at once proceed to Dunster, both to be out of the way of the epidemic, and to encourage the new levies, little thinking that "the disease was as hot at Dunster town, just under the castle." Savage writes, "there were several entries of the burials of soldiers from the castle." (1645.) To corroborate this statement, we searched the defoliated register of the parish. From this record, which unfortunately became imperfect at the end of June, 1645, and contained on the same page, where the burials for that year were written, some which took place several years later, we discovered that *four* soldiers only were buried in the first half year of 1645, two in February, one in March, and another in April. These must be "the several entries" alluded to by Savage. In the preceding two years the number of deaths recorded was, in 1643, 27, and in 1644, 42; whilst in the first half of the pestilential year, 1645,

58 were buried. In May alone 23 died, which nearly equals the full amount of the whole of 1643. The plague seems to have made a sudden leap in May, the registrations for each month being—in January, 8 ; February, 5 ; March, 2 ; April, 7 ; May, 23 ; June, 13 : in all—males, 30 ; females, 28 ; total, 58.

It can easily be imagined that the plague found an appropriate nidus in this little village, when we consider that even up to a late date there were open ditches running down through the main streets, into which filth of all kinds was thrown. On each side of these streets were high causeways for foot passengers, which so narrowed them as to admit of but one vehicle passing at a time. There were also two large dungheaps—one in the middle of the town, and the other just above the principal inn. What is now an open street, just below the market cross, was filled up by the market place and town hall, making the streets on each side both close and narrow.

It was in the long street, called West Street, over-shadowed by wooded hills and the towers of the castle, that the plague was most virulent ; and it was this street where the peculiar sanitary regulation was carried out which so much interested us. It appears that the people, panic-struck by the falling of the victims of the plague around them, and convinced that the epidemic was in the air, made an attempt to shut the enemy out, as they fancied. This they did by entering into an arrangement one with another to make all their houses communicate, so as to avoid the necessity of going into the street for the various necessaries of life. Doors were made between the different tradesmen's houses, so that in the longest street one could walk from one end to the other without going into the street. Some of these doors still remain, and tell their interesting tale. One we remarked especially perfect, in Mr. Nation's house, which was formerly in the possession of the father of Mr. Withycombe, who obligingly afforded us all the information in his power.

We have calculated that the average mortality in Dunster is 24 in the year, which statistic we have arrived at by taking the number of deaths which occurred in eighteen different years taken promiscuously from the year 1575 to the year 1805.

The greatest number recorded was in the years 1645 and 1697, in which last there were registered 86 burials without any cause assigned for this excess in mortality. Had the number in the second half of 1645 equalled that of the first, that for the whole year would have been nearly five times greater than the average: a curious coincidence, when we remember that Minehead suffered in the same ratio in the same year.

*A. Haviland*

